# A qualitative evaluation of participants' experiences of using co‐design to develop a collective leadership educational intervention for health‐care teams

**DOI:** 10.1111/hex.13002

**Published:** 2020-01-30

**Authors:** Kirsten Siig Pallesen, Lisa Rogers, Sabrina Anjara, Aoife De Brún, Eilish McAuliffe

**Affiliations:** ^1^ UCD Centre for Interdisciplinary Research, Education and Innovation in Health Systems, School of Nursing, Midwifery and Health Systems University College Dublin Dublin 4 Ireland

**Keywords:** co‐design, health services research, implementation science, participatory design, qualitative research, quality improvement, stakeholder participation

## Abstract

**Introduction:**

Co‐design involves stakeholders as design partners to ensure a better fit to user needs. Many benefits of involving stakeholders in design processes have been proposed; however, few studies have evaluated participants’ experience of co‐design in the development of educational interventions. As part of a larger study, health‐care professionals, researchers and patients co‐designed a collective leadership intervention for health‐care teams. This study evaluated their experiences of the co‐design process.

**Methods:**

Semi‐structured interviews were conducted with individuals (n = 10) who took part in the co‐design workshops. Interviews were audio‐recorded, transcribed verbatim and analysed thematically.

**Results:**

Four key themes were identified from the data: (a) Managing expectations in an open‐ended process; (b) Establishing a positive team climate; (c) Focusing on frustrations—challenging but informative; and (d) Achieving a genuine co‐design partnership.

**Conclusions:**

The development of a positive team climate is essential to the co‐design process. Organizers should focus on building strong working relationships from the beginning to enable open discussion. Organizers of co‐design should be conscious of establishing and maintaining a genuine partnership where participants are involved as equal partners and co‐creators. This can be done through the continuous use of feedback to allow participants to influence the workshop directions, and through limiting researcher domination. Lastly, co‐design can be daunting, but organizers can positively impact participants’ experience by acknowledging the emergent nature of the process in order to reduce participant apprehension, thereby limiting the barriers to participation.

## 1 INTRODUCTION

Involving stakeholders in service development has been shown to lead to better idea development, enhanced alignment to user needs, greater service user satisfaction^1^ and improved stakeholder ownership of health‐care initiatives.^2^ In health care, patient/public involvement (PPI) has been proposed to result in benefits such as improvements in staff and patient morale and the development of services that respond better to community needs.^3^ PPI in research is advocated both on pragmatic and on moral grounds,^4^ and there is a growing call from patient communities to be involved in all aspects of health policymaking, clinical care and research.^5^

Co‐design approaches use the real‐life experience of patients and health‐care providers (HCPs) to improve service design and delivery.^6^ Co‐design goes beyond user involvement, where end‐users have a consultant or advisory role.^7^ In co‐design, stakeholders are involved as equal partners and co‐creators, and the experiences of users and communities are at the core of the design process.^8^, ^9^ Many benefits of PPI and participatory design have been identified in the literature, such as the ability to capture experiences of patients and HCPs, ensuring that researchers, leaders and policymakers understand the reality and challenges faced by service users and deliverers.^3^, ^9^ It is widely acknowledged that the uptake of evidence‐based health‐care interventions is challenging^10^, ^11^, ^12^, ^13^ and that HCPs can be instrumental determinants in impeding the change process.^14^ By using a more ‘bottom‐up’ approach, local needs and concerns are reflected, building stakeholder commitment, subsequently improving the likelihood of implementation success.^2^, ^15^

Few studies have evaluated stakeholders’ experience of participating in co‐design. Maher et al^16^ evaluated users’ experience of co‐design from a practical perspective, identifying challenges and solutions to engaging patients. Bowen et al^17^ evaluated participants’ experience of a participatory health service design process and found that the methods applied during the process, such as story‐sharing and emotional mapping, were effective in establishing working relationships among the participants involved. More recently, Haines et al^18^ evaluated participants’ experience of co‐designing a peer support model and found that patients and families appreciated feeling valued and heard and that clinicians found the approach beneficial. Previous studies of co‐design experiences have applied the NHS experience‐based co‐design (EBCD) framework, a structured, stepwise approach that uses storytelling to gather patient and staff experiences to identify opportunities for improvements and inform co‐design groups working on the identified areas.^19^, ^20^

**Figure 1:**
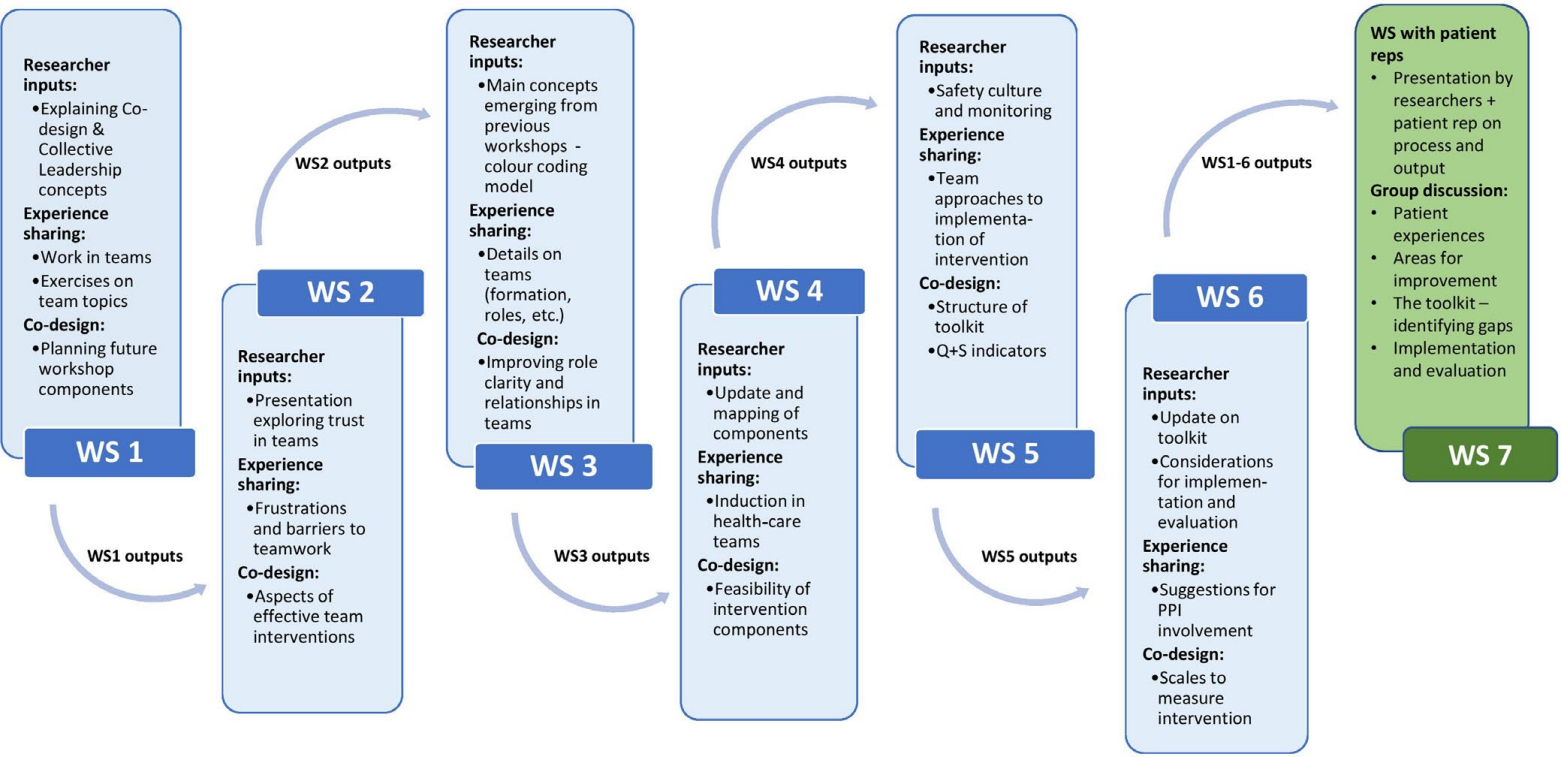
Diagram of the applied co‐design approach. *Note*: Each workshop consisted of experience sharing, input from researchers (eg background information or evidence synthesis) and a co‐design piece where team members would work to design specific intervention components. Each workshop would be informed by topics raised, issues, discussions and suggestions from previous workshops

The co‐design process evaluated in this study is part of a larger research programme on Collective Leadership and Safety Culture (Co‐Lead), which aims to introduce collective leadership to health‐care teams to improve team performance and patient safety culture.^21^ The objective of the co‐design process was to produce a team‐based intervention to develop collective leadership competencies within health‐care teams. Introducing a leadership model through team‐based training differs from traditional leadership development approaches, and there is little existing knowledge on how best to deliver such an intervention. Therefore, co‐design was applied to construct a more needs‐based authentic solution and enhance ownership of the intervention to increase the prospect of successful implementation. The process entailed six workshops, each consisting of introductions, researcher inputs, experience sharing and a co‐design piece (Figure 1). The workshop organizers strived for collective leadership to be practised within the co‐design team, and interactive exercises such as word association exercises and implementation road maps were used to facilitate the process. A summary of the workshops can be found in Appendix S1. For a more detailed description of the co‐design process, see Ward et al^22^

The process resulted in a toolkit of team training sessions to be self‐facilitated by health‐care teams on a monthly basis (Table 1). This intervention is currently under evaluation.

**Table 1:** Components of the co‐designed team‐based educational intervention

	Co‐lead components
Foundational components (mandatory)	1. Team Values, Vision and Mission
	2. Team Goal Setting
	3. Role Clarity
	4. Collective Leadership for Safety Skills
	5. Risk and Safety Management at the Team Level
	6. Monitoring and Communicating Safety at Team level
Targeted components (to be selected by teams based on their needs/priorities)	7. Effective Team Meetings
	8. Removing Frustrations/Blockers
	9. Building Trust
	10. Structured Interdisciplinary Rounds
	11. Challenging Unsafe Behaviours
	12. Communication at Safety Critical Moments
	13. Talking about Safety (PlayDecide game^30^)
	14. Safety Pause Huddles
	15. High Reliability at the Team Level
	16. Developing a positive work environment
	17. Emotional Support in Teams
	18. Enhancing Person‐Centred Care
	19. Sustaining Improvements

This study aims to evaluate how the co‐design process was experienced by those involved and to offer recommendations to researchers based on these experiences to inform future co‐design processes. This study differs from previously reported studies as it attempts to embody a collective leadership approach within the process, resulting in a less‐directed approach and an emergent rather than a pre‐determined structure. Furthermore, the designed ‘product’ is an educational intervention for health‐care professionals rather than a patient service. Both the structure and aim are therefore novel. As health‐care professionals are both designers and the target audience for the intervention, it is important to explore their experiences alongside those of the researchers and patient representative, allowing for comparisons of how the process was experienced differently by members of the co‐design team.

## 2 MATERIALS AND METHODS

### 2.1 Participants

Twenty‐one people took part in the co‐design process (7 researchers, 12 HCPs and a patient representative). Researchers were members of the Co‐Lead research programme who would be involved in implementing and evaluating the intervention and brought their experience of leadership and patient safety to the co‐design process. Two HCPs had national roles as quality and safety advisers. The rest represented the teams participating in the pilot implementation of the co‐designed intervention. Teams selected volunteers who could represent their needs, priorities and experiences. One patient representative was recruited through a hospital patient liaison service. Another was invited to take part as a member of a national patient safety organization, but was prevented from participating by illness. Therefore, a seventh workshop was held with five patient representatives and advocates to ensure additional PPI input. Participants who attended minimum two workshops were invited to take part in the evaluation. Of the 13 who were invited, 10 consented to take part. Participant characteristics are presented in Table 2.

**Table 2:** List of participant characteristics (n = 10)

Participant	Gender	professional role	Attendance (no. of workshops)
HCP1	F	Business Manager	5
HCP2	M	Consultant	5
HCP3	F	Nurse Manager	6
Patient Rep	M	Patient Representative	6
Researcher 1	F	Experienced Health Systems Researcher	3
Researcher 2	F	Experienced Health Systems Researcher	6
Researcher 3	F	Experienced Health Systems Researcher	6
Researcher 4	F	Research Team Member	6
Researcher 5	F	Research Team Member	5
HCP/Researcher	F	Research Team Member and Hospital Manager	6

### 2.2 Data collection

Semi‐structured interviews were conducted with all participants, nine face‐to‐face and one via telephone (mean duration 31 minutes, range 19‐46 minutes), between March and August 2018 by two researchers who had not been involved in the co‐design workshops. Questions pertained to participants’ expectations for, and experiences of the co‐design process, including positive aspects and challenges, workshop content, the decision‐making process, and perceived learning and impact. The topic guide is available in Appendix S2.

Written feedback, in the form of anonymous feedback forms distributed at the end of each of the first five workshops, was analysed alongside the interview data. The feedback forms (Appendix S3) contained closed evaluation questions accompanied by open fields for writing additional/explanatory comments.

### 2.3 Data analysis

Interviews were audio‐recorded and transcribed verbatim with all identifying information removed. Two researchers (KP and LR) who had not been involved in the co‐design workshops evaluated the interview data using thematic analysis, as outlined by Braun and Clarke.^23^ The initial coding was conducted independently by each researcher, using NVivo 11^24^ to organize and manage the data. Themes were identified, reviewed and refined through researcher discussion and agreement. The analysis was further refined through discussions with co‐authors throughout the process.

### 2.4 Ethical approval

The study received ethical approval from [redacted for peer review].

## 3 RESULTS

Four key themes were identified from the data: (a) Managing expectations in an open‐ended process; (b) Establishing a positive team climate; (c) Focusing on frustrations—challenging but informative; and (d) Achieving a genuine co‐design partnership.

### 3.1 Managing expectations in an open‐ended process

Many participants experienced apprehension and uncertainty ahead of the workshops, and most reported that they had no previous experience with the co‐design format.

#### 3.1.1 Ability to manage/contribute in a process that is unfamiliar

Both researchers and HCPs recalled having feelings of apprehension ahead of the first workshop. Some researchers reported feeling ‘*terrified’* (Researcher 2) and anticipated that the process can ‘*be uncomfortable’* (Researcher 3) or ‘*provoke anxiety’* (Researcher 1), as it involves starting from a blank slate. One researcher (HCP/researcher), who was new to the research team, reported feeling nervous regarding her colleagues’ ability to facilitate the sessions as this required a different skillset. Another (Researcher 5) mentioned that while the research team had anticipated that the co‐design process would be difficult, HCPs might not have expected the associated level of complexity. One researcher reflected that the process could have been improved by the researchers highlighting the complexities of co‐design in advance:

I think if I was using that process again with people, I would always begin the sessions with “you are going to be uncomfortable, this is going to be difficult” (HCP/Researcher)

The HCPs’ apprehension ahead of the first workshop was centred around not knowing what the process would entail, and doubts about their own ability to contribute:

‘I suppose you go in a little bit anxious. (...) Would you have sufficient knowledge, skills and knowledge to contribute to it?’ (HCP1)

All HCPs reported that it took a few workshops for them to fully understand what co‐design was about, with one HCP describing how she was ‘*at sea’* (HCP1) for the first few workshops. Another (HCP2) reflected that the initial uncertainty ‘*was part of the process, it had to be there, not a bad thing’*. Researchers acknowledged that there was a lot for the non‐researchers to comprehend.

However, the anonymous feedback after the workshops does not confirm this general uncertainty. Following the first workshop, all participants (n = 14) reported that they understood the aims of the research programme and the purpose of the co‐design phase, ‘*great teamwork in explaining programme’*, ‘*explained very well, good understanding of the programme’*. Another respondent suggested a bit more on‐going uncertainty, describing their understanding as ‘*work in progress’*.

Participants only described feeling apprehensive during the early workshops, which suggests that developing familiarity with the process and contents reassured them. As co‐design is an open‐ended and iterative approach, discussion points may be raised multiple times. The patient representative mentioned that the group was not ‘*making progress in the way [he] thought would be made’.* This suggests that not all participants were anticipating outputs to be emerging slowly. Some non‐research participants described perceiving a sense of progress and direction as the toolkit began to emerge, indicating that much of the discomfort may have related to the open‐ended nature of the process.

#### 3.1.2 Working with people from different backgrounds

Researchers and HCPs described the interaction with other professions as initially daunting. There was a perception among some HCPs and researchers that other co‐design team members were more highly qualified than themselves. However, this sentiment was limited to the beginning of the process.

It’s daunting to sit around with all of these academic people, and they’re talking about theirbackground and their degrees, and you feel like you're the nurse on the ground (HCP3)

I have a thing about HCPs being way more professional than me (…) I think that just made me nervous (Researcher 5)

The patient representative described himself as ‘*brave’* but acknowledged that he initially felt *‘out of [his] depth’* as other participants had a health‐care qualification or academic degree. One HCP (HCP3) described initial apprehension about working with a patient representative, but quickly realized her concerns had been unfounded.

### 3.2 Establishing a positive team climate

Despite this initial uncertainty, all participants reported enjoying the experience and atmosphere within the co‐design team. Engagement was described as high throughout the process.

‘I think it was a fantastic workshop (…) everybody engaged that was involved’ (HCP1)

Participants associated working in small, changing groups with establishing a positive team climate. Some mentioned the importance of small breaks for social interaction, as it allowed participants to build relationships in an informal setting. The researchers strived to limit any hierarchy or top‐down decision making, which was acknowledged by participants who reported collaborative decision making with ‘*no real decisions made from the top of the table’* (Patient Rep).

Participants described a sense of openness within the group, enabling everyone to speak and contribute. This was attributed to the way the sessions were facilitated and to the team atmosphere formed by the individuals involved. The patient representative recognized that they practised collective leadership within the co‐design team, noting how he realized that ‘*the model [they] were trying to create actually turned out to be the model of what [they] were doing’*. Participants generally described the co‐design team as easy to get on with, with no strong or difficult personalities. One researcher (Researcher 4) suggested that participants were too positive, reflecting that it could be beneficial to include participants who were more critical to gain a different perspective.

Several participants mentioned that the trust between team members grew and that they became ‘*more familiar and relaxed’* (HCP1) as the workshops progressed, which enabled better discussions and teamwork. Two HCPs noted how it was easier to build relationships with the regular attendees.

### 3.3 Focusing on frustrations—challenging but informative

Most researchers reported experiencing a ‘*low’* (Researcher 5) or ‘*dip in energy’* (Researcher 2) at the second workshop where participants were asked to reflect on barriers to effective teamwork. Some researchers described how this affected the energy in the room:

I suppose in a sense I had a vibe that this was unachievable (…) I was picking that up, I suppose, from the energy in the room (Researcher 4)

However, only researchers reported experiencing this ‘*dip’*, which might indicate that although the emphasis on barriers caused a negative focus during the workshop, non‐researchers found the discussions necessary and relevant. Some researchers acknowledged that this ‘*low’* generated important material and that it promoted a shared understanding of the difficulties associated with implementing research into routine practice.

In the anonymous session evaluations, all participants (n = 14) reported that the second session was worth attending and that they understood how the workshop components fit with the co‐design process. Some comments did indicate that the session highlighted the challenges of the task. However, most comments were focused on how these difficult discussions generated valuable learning and outcomes. Some feedback was decidedly positive, with participants describing ‘*excellent engagement and discussion’* and that ‘*meetings are very positive’.* Some also mentioned the benefits of sharing experiences as ‘*important to realise that challenges we have are similar to others’.*

### 3.4 Achieving a genuine co‐design partnership

Researchers were conscious of allowing participants to influence the workshop contents, avoiding excessive researcher contribution, and HCPs recognized that they had impacted the direction of the workshops. Some researchers experienced that ceding control to achieve this was unfamiliar and at times uncomfortable.

#### 3.4.1 Value of feedback

To allow participants to influence the direction of the workshops, the research team acquired feedback throughout the process through open discussions and anonymous evaluation forms after each session. Participants noted how eliciting opinions from the co‐design team allowed members to influence the workshop contents, impacting their overall direction:

…{researchers} might say “this has come up a lot today, I think we’ll talk about this at the next meeting” and then that’s how the next meeting might have even been decided on… (HCP3)

This was recognized by many as a beneficial method of ensuring the relevance of workshop content. HCPs highlighted the importance of the workshop topics coming from ‘*people on the ground with real life experience’* (HCP3). One researcher reflected on whether participants were sufficiently involved in setting the agenda, reflecting that it might not have been ‘*true co‐design’.* This view, however, was not reported by others. One HCP described the risk of ‘*do[ing] it {co‐design} for the optics but not necessarily get[ting] the throughput of it’* (HCP2), but he did not associate this with his experience of the process.

#### 3.4.2 Sharing control and power

Using participants’ feedback, the researchers regularly went ‘*back to the drawing board’* (Researcher 2) to allow participants to influence the workshop direction. This iterative process was acknowledged by one participant (HCP/Researcher) as ‘*very brave’*. One researcher described the workshops as ‘*stressful’* (Researcher 2), as they had to surrender control and ‘*wait to hear what [non‐researcher participants] come up with’*, while another described how the nature of co‐design made it hard to pre‐plan workshop content.

To achieve a genuine co‐design partnership, researchers described being conscious of sitting back and allowing content to emerge from the other participants, including ‘*stepping out and not being there for all the sessions’* (Researcher 1). Some found this ‘*difficult’* (Researcher 1) or ‘*uncomfortable’* (HCP/Researcher) as they found themselves in an unfamiliar role. However, one researcher in particular reported great learning from taking on this unfamiliar role, which she has applied her regular work:

I just learned a huge amount from letting it happen and by giving that autonomy and empowering people in the process (…) I don’t always have to be doing the talking (HCP/Researcher)

## 4 DISCUSSION

This study explored participant experiences of co‐designing a collective leadership educational intervention for health‐care teams. It found that increased levels of trust and the development of relationships between participants were key to enhancing teamwork and the psychological safety of participants, which stimulated their contribution during the process. This was facilitated by group work and interactive exercises. Organizers sought to inhibit hierarchy and support the formation of relationships by providing opportunity for informal communication and relationship building. The co‐design process resulted in the successful development of an educational intervention, and the results of a pilot evaluation are currently being prepared prior to a planned large‐scale evaluation.

The co‐design aim was to produce a collective leadership intervention for health‐care teams. According to Tuckman's theory of group development,^25^ any newly established team is strongly reliant on leadership to provide clear direction and establish goals. Despite the intentional absence of organizer‐driven direction (in keeping with a collective leadership approach), the co‐design team progressed to produce outputs without the commonly experienced early team struggles or disagreements said to be an inevitable part of team formation.^25^ In contrast, Bowen et al^17^ reported both initial tension and conflicts throughout their co‐design process. While team members are expected to have different values, needs, and competing priorities regarding their involvement,^26^ co‐design seeks to overcome potentially diverse priorities in order to achieve an agreed solution that will benefit all. The absence of conflict or disagreement in this co‐design process might be in part due to a concerted effort to allow space and time for participants to voice their opinions from an early stage in the process, and the consistent messaging from the researchers that they would not be taking a leadership role in the co‐design process. An open and supportive team climate was established early in the process through these efforts to model collective leadership within the co‐design team. Furthermore, the participants were described as an ‘*easy [group] to get on with’* (Patient Rep) with no strong or difficult personalities. These factors appeared to result in a cohesive group with a common sense of direction, which may have mitigated against the risks relating to differing and competing priorities among co‐designers.^26^ The team atmosphere and the practice of collective leadership ensured that the representation of diverse viewpoints was an asset, allowing for the design of an intervention that addressed a variety of needs and concerns.

The practice of collective leadership may have nurtured some of the team features that characterize a positive team climate,^27^ which is associated with team innovation and creativity.^28^, ^29^ This was further facilitated through the structure and organization of the workshops. Group work, movement and interactive exercises, as well as breaks for social interaction, were considered important methods to enable the formation of relationships, breaking down barriers and enhancing trust between participants. Some participants described finding it easier to engage with regular attendees than with those who only attended a few workshops. This suggests that the development of relationships between participants was crucial to the engagement and atmosphere within the group and that a relatively stable group of participants may contribute to the establishment of a positive team climate. Importantly, participants reported influencing the decision making and feeling able to contribute in a non‐threatening environment. West^28^ proposes that these characteristics, collectively named participative safety, lead to a higher investment in outcomes of decision making and increased innovative contributions from participants. Thus, the organizers’ time investment in achieving collective leadership and supporting the early formation of trusting relationships not only enhanced participants’ experience of the process, but was vital in facilitating innovative contributions and ownership.

The establishment of participative safety and a positive team climate is important in encouraging participants to share stories and experiences. Eliciting experiences is a crucial part of co‐design and has previously been described as important in creating an alliance of change between HCPs and patients.^17^ Our evaluation has highlighted how storytelling can impact individuals differently within the co‐design team. This is most clearly demonstrated by the second workshop where researchers reported experiencing a ‘*dip in energy’*, which no non‐researcher participants experienced. Our interpretation is that the researchers’ experience resulted from hearing the current frustrations of teams and realizing the enormity of the challenge ahead: getting teams to function collectively. In contrast, for HCPs, the session was likely a cathartic experience, allowing a safe space to discuss their frustrations in a manner that was not possible within their regular work environment.

Most participants reported experiencing apprehension and uncertainty before and during the early workshops. Some initial tension and apprehension among participants was similarly noted by Bowen et al^17^ However, the sense of anxiety recalled by some participants in this study appears more significant than previously reported. This might be because the purpose of the process was to design an educational intervention, which is usually perceived as the role of universities. For Bowen et al,^17^ the objective was to design a quality improvement intervention within the health service, and thus, the expertise lay clearly with the HCPs. In our co‐design process, stakeholders were asked to contribute in an area that would traditionally be considered academic territory, which might have caused anxiety among non‐researcher participants. Furthermore, few participants had any prior experience working with co‐design, which differs from many other team processes due to its open‐ended nature, and the practice of collective leadership was unfamiliar to most participants. This may have contributed to their early apprehension and discomfort with the process. For the researchers, a clear source of apprehension was the open‐ended nature of the co‐design process, which posed a challenge in organizing and preparing the workshops. Our findings suggest that organizers could improve participants’ experience by clarifying early on that uncertainty is an inevitable and necessary part of the co‐design process. This clarification should be focused on anticipating and accepting the uncertainty, rather than avoiding it. The sense of apprehension and uncertainty is not found in the feedback forms from the individual co‐design workshops, suggesting that the uncertainty might have been related to the overall process, as participants reported experiencing each workshop as meaningful in addressing the team's goal.

The concern relating to one's ability to contribute will likely pose a barrier to participation for some individuals. For example, the patient representative described himself as a ‘*brave person’*; yet found himself feeling ‘*out of [his] depth’* initially. If less confident individuals are reluctant to participate due to apprehension or uncertainty, important voices might be lost. It is important to accurately manage participants’ expectations so uncertainty does not become frustration and discouragement. In their evaluation of participants’ experience and engagement in collaborative design, Maher et al^16^ found that participants experienced feelings of frustration and dismissal due to lack of information about progress and outcomes. In our study, some participants reported that their feelings of uncertainty were diminished as the intervention began to emerge. The patient representative's frustration with an apparent lack of progress was likely also due to inadequate visibility of the progress made. This suggests that on‐going awareness of the progress and output is important in enhancing participants’ experience. Thus, early and continuous information about the process, the expectations and the progress made are key in establishing and maintaining stakeholder engagement and involvement throughout the co‐design process.

In co‐design, it is important that stakeholders are involved as equal partners and co‐creators.^16^ Maintaining this approach is a delicate balance, considering the time pressures on participants and the importance of efficiently using time to develop outputs that reflect the expertise and experience of the group. The high number of participating researchers in the co‐design presented a potential challenge in achieving and maintaining this balance. Therefore, researchers were conscious of refraining from dominating discussions, and attendance numbers were pre‐planned to avoid a majority of researchers. In our co‐design process, non‐researcher participants reported that they were listened to and influenced the direction of the workshops, suggesting that the balance was successfully maintained. However, one researcher felt that there was insufficient opportunity for participants to set the agenda. Although no other participants reported this, co‐design organizers should be aware of this concern. For example, Bowen et al^17^ found that participants did not see themselves as co‐designers, but rather as having a consultant role where they would share their experiences, and the researchers would design the service. Their co‐design process followed the NHS EBCD model, which is a more structured and defined approach compared to the one evaluated in this study. The more organic approach adopted here, one which focused on practicing collective leadership in the team, might have enhanced the participants’ sense of ownership. Haines et al^18^ found that some participants struggled to progress past storytelling and contribute in the more task‐driven design aspects. This was not observed in our co‐design process. Our findings suggest that although a less structured approach may initially be more uncomfortable, it might positively affect the formation of a genuine co‐design partnership.

Regardless of the approach, it is important that organizers are conscious of achieving and maintaining a genuine partnership, allowing all stakeholders equal opportunity to influence the design output. Failing to do so would violate the principles underpinning co‐design and risk tokenistic stakeholder involvement, which is both ethically problematic and fails to profit from the benefits of true co‐design. It is essential that organizers not only request feedback for evaluation purposes, but continuously elicit and actively use feedback and discussion topics to set the agenda for the process. We therefore advocate for the inclusion of representatives from stakeholder groups in planning and organizing the process to ensure that the researchers/organizers do not disproportionately affect the workshop direction and output. Furthermore, it would help to ensure that the work commitments of involved groups are considered. It is also important to reflect on the effect of the team composition on the establishment of a co‐design partnership. Unequal stakeholder representation could impact the balance and power relations in the group, negatively affecting participants’ willingness and ability to contribute. Despite having only one patient representative, this was not observed in our co‐design process, which might be due to this individual's confident character and the absence of strong personalities in the group. Organizers should endeavour to obtain adequate and equal representation to ensure that all stakeholders are able to make their voices heard and influence the co‐design process.

This study contributes to the literature on co‐design by evaluating participants’ experience of a novel co‐design approach and objective. Our findings have led to the provision of recommendations to guide researchers and organizers of co‐design projects (Table 3).

**Table 3:** Recommendations for organizers of co‐design processes

Practical recommendations	Scheduling	Participants reported half‐day workshops once a month as suitable. Longer workshop duration might have decreased participant focus
	Location	It is important that the co‐design workshops take place in a location away from participants’ workplace. This allows for protected time and neutrality for all participants. A university meeting room was deemed appropriate by all participants
	Stakeholder involvement in planning	It might be useful to involve stakeholders in the organization and preparation of workshops to ensure that any limitations due to participants’ work circumstances are adequately considered, and to limit excessive researcher input
	Participant preparation	Participant preparation in advance of meetings should be limited. Dedicated time should be allowed at workshops for participants to read essential material, rather than expecting participants to read material in advance. However, a team homework exercise was accepted by participants and perceived as beneficial by researchers, so carefully selected, relevant, *volunteer* preparation might be appropriate
Supporting the formation of a positive work climate	Workshop content	Workshops should include work in small and frequently changing groups to encourage participant interaction and the formation of relationships
		All workshops should include interactive exercises and movement to put participants at ease. This is particularly important during the first workshops when relationships have not yet been formed
	Informal talks	Short coffee breaks are encouraged to allow participants to network and form relationships in an informal manner.
	Attendance	Consistent attendance is essential for the formation of good working relationships. Co‐design members joining the process late should be limited/avoided unless suggested as necessary by the team to ensure appropriate representation
	Promote equality	Organizers should strive to limit any group hierarchy, for example by encouraging the use of first names rather than titles
Enhancing participant experience	Manage expectations	In order to reduce participant apprehension, organizers should make an effort to manage participants’ expectations when inviting them to take part, in order to avoid any early‐stage anxiety or apprehension. This could be achieved by explaining the co‐design process, highlighting the method's emergent nature, and emphasizing that uncertainty is a necessary and inevitable part of the process
	Review progress	Organizers should make regular updates/progress reviews, in order to make explicit the team's progress towards the goals. Lack of awareness of progress might lead to feelings of discouragement, which might adversely affect team engagement
Ensuring a genuine co‐design partnership	Allow participants to influence contents	Organizers should make sure to collect and use participant feedback to inform the direction of the co‐design process. Facilitating the collection of verbal and anonymous written feedback enables an open and honest atmosphere. Furthermore, topics raised by participants during workshops should be developed further by the organizers and followed up at subsequent workshops
	Ensure sufficient patient representation	Organizers should consider the risk of patient attrition when recruiting participants to ensure that the patient voice is sufficiently represented. Although not observed in the current study, failing to include patient representatives in sufficient numbers might negatively impact on their psychological safety and their ability to engage in an equal partnership
	Limit excessive researcher input	Organizers/researchers should be prepared to take on a more observatory role during workshops, to allow for ideas to emerge from the participants. Involving external stakeholders in the organization and preparation of workshops might help facilitate this

### 4.1 Strengths and limitations

In order to limit researcher bias, the interviews and data analysis were carried out by researchers who were not involved in the co‐design process. Initial coding was conducted independently by two researchers, and audit trails were kept throughout the coding process to ensure transparency of the analysis.

The results reflect the experiences of a group of co‐design participants; however, multiple factors limit the generalizability of findings. The interview sample consisted of people who had participated in most workshops, suggesting that they might have been participants who enjoyed the workshops or appreciated the benefits of co‐design. Only one patient was involved throughout the co‐design process. As key objectives of Co‐Lead are improved care and patient safety, a higher representation of patients would have provided further insight. Additionally, the voice of the HCPs is under‐represented in the interview sample. Finally, the interviews took place a year following the workshops, which might have led to participant recall bias. The study has attempted to account for this by using workshop feedback to include participants’ real‐time experiences.

## 5 CONCLUSION

This study contributes to the literature by drawing on co‐design participants’ experience to inform recommendations for future co‐design processes. Our findings demonstrate that early efforts to build trusting working relationships and promote collective leadership help to enable the participative safety required to ensure innovative contribution and ownership from co‐design participants. Co‐design organizers should be conscious of establishing and maintaining a genuine partnership with stakeholders involved as equal partners and co‐creators. In this co‐design process, this was achieved through the continuous use of feedback, allowing the participants to influence the workshop direction, and through limiting excessive researcher input. Lastly, co‐design can be daunting. Organizers can positively impact participants’ experience by addressing its emergent nature, and the associated uncertainty, in order to reduce participant apprehension, thereby limiting the barriers to participation.

## CONFLICT OF INTEREST

The authors declare no conflicts of interest.

## Data Availability

Interview data are not publicly available due to their containing information that could compromise the privacy of research participants.
