# Supplementary material for: A qualitative evaluation of participants' experiences of using co‐design to develop a collective leadership educational intervention for health‐care teams
Source: Health Expect. 2020 Jan 30;23(2):358–67. doi: 10.1111/hex.13002 (PMC7104638; doi:10.1111/hex.13002)
Supplement: Supplementary file 1 [file HEX-23-358-s001.docx]

**Appendix A – Summary of co-design workshops**

|  | Inputs | Exercises |
| --- | --- | --- |
| Workshop 1 (3 hours) | **External stakeholders:** Healthcare professionals presented on the work in their teams  **Researchers:** Researchers presented on Co-Lead programme and co-design as a method. | Introductions.  Word association exercises, group exercises and pair discussions re. team performance and patient safety.  Pair discussion on planning components of future workshops |
| Workshop 2 (3 hours) | **External stakeholders:** Assisted reflection on previous experiences of frustrations related to teamwork.  Team members were asked to read an article on overcoming barriers to effective teamwork in healthcare.  **Researchers:** Researchers did a presentation on trust, based on discussions in the first workshop. | Team members fed back on the reflection exercise through pair discussion, stickies-exercise on barriers and main group feedback.  Word association exercise related to building trust.   Small group discussion on case study of effective team interventions to identify useful aspects to incorporate into the co-design intervention. |
| Workshop 3 (3 hours) | **External stakeholders:**  As homework, healthcare teams were given a template in which to fill details on their own teams (formation, roles and responsibilities, etc.).  **Researchers:** Researchers presented suggestion to colour code the intervention according to three main concepts: collective leadership, team performance and safety culture. | Teams presented details on their teams using the PowerPoint template.  Exercises to develop intervention to improve role clarity and relationships in teams |
| Workshop 4 (3 hours) | **Healthcare Team members:** Teams were tasked with developing an ‘induction pack’ to induct new people onto the team  **Researchers:** Update on work to date, incl. revised models previously presented. Mapping **Core Co-Lead concepts** onto **Behaviours** that we desire to impact onto **Intervention components** to introduce the changes. | Discussion of main Co-Lead concepts and their relation to team behaviours, e.g. what behaviours would be seen in a team with Collective Leadership? How could these behaviours be achieved?   Discussion exercises about collective leadership interventions and their feasibility in the teams represented in the co-design group. |
| Workshop 5 (3 hours)_ | **External stakeholders**: Teams asked to consider the development of the intervention to date, how they would implement it with their team.   Presentation by healthcare professional with role in Q&S made a presentation on safety culture.   **Researchers:** Presentation on the behaviours related to safety culture and how to achieve them, and on monitoring patient safety at team level. | One team presented interventions they felt their team needed  Exercise on co-designing team-level Q&S indicators.  The draft toolkit (overall structure) was presented and discussed. Two interventions (Role Clarity and Team induction) were discussed in more detail. |
| Workshop 6 (all day) | **Healthcare Team members:** Presentation on current status of the intervention and discussion on implementing the intervention.  Presentation and discussion on the suggested evaluation approach and scales.  Discussion around involvement of patient representatives in the process and how only one patient representative made the workshops.  A spoken and written evaluation was carried out of the process. | Each person was asked to review aspects related to the content and feasibility of the intervention components.   Exercise to decide on scales to measure core components.   Discussion on how to get further PPI feedback on the intervention toolkit |
| Workshop with Patient Representatives & Advocates | Presentation by researchers and the patient representative who had participated in the co-design process on the process of designing the intervention and the intervention components that had been designed. | Group discussions on experiences of healthcare teams in hospitals or other healthcare settings and the impact of the team’s functioning.   Group discussion on the intervention toolkit pieces and any gaps, as well as the implementation and evaluation of the toolkit |

**Source: Ward et al.**^22^
