# Supplementary material for: A qualitative evaluation of participants' experiences of using co‐design to develop a collective leadership educational intervention for health‐care teams
Source: Health Expect. 2020 Jan 30;23(2):358–67. doi: 10.1111/hex.13002 (PMC7104638; doi:10.1111/hex.13002)
Supplement: Supplementary file 2 [file HEX-23-358-s002.docx]

**Appendix B – topic guide for interviews**

Introduce self

Explain purpose of the interview

Request permission to audio-record

Answer any questions from participants; explain right to withdraw

**Background Questions**

1. Can you tell me a bit about your role in the health service/research team? How long have you been in this role
2. How did you first hear about the Co-Lead project and the co-design team?
3. What made you decide to be involved?
4. Why do you think you were asked to take part? What were your expectations?
5. Before starting the project what was your understanding of co-design?

- What did the word co-design mean to you?
- Have you worked this way before?
- If this process is different from how you usually work, how is it different?

1. Were you familiar with the term “Collective Leadership” before the workshops?

**Experiences**

1. On arriving to UCD for the 1^st^ day of the co-design workshops, when you walked into the boardroom, can you remember how you were feeling? {If needed, why do you think you felt that way?}
2. What were your expectations?
3. Did you know anyone else in the room?
4. How did you get to know each other?
5. Co-lead held 6 workshops from January to June 2017; do you know how many you attended?
6. Could you take me through the co-design process?

- What happened during workshops?
- What was discussed in the first session/what did the first session consist of?

1. Could you describe what worked well during the co-design process?

- What did aspects did you enjoy/find interesting or useful?
- Did the half day suit? Was it long enough?
- Was the venue a suitable place to come together?

1. What aspects do you think didn’t work so well?

- Were there any challenges? If so, what were they and what steps were taken to overcome these challenges?

1. How were decisions made in the workshops?

- Did you feel that you could contribute your opinion to discussions?
- Do you think everyone was comfortable contributing? Why?

1. Do you feel the topics discussed at each workshop reflected the goals of the team?

- Were the topics relevant?
- Were the topics important?

1. Did you feel you could add suggestions about what should be discussed at the workshops?
2. Would you have liked to be involved in running any of the workshops?

- Why/Why not?

1. What do you think you have brought to the co-design process?

- What were the specific qualities that you contributed to the discussion?

1. Do you think everyone was engaged in the workshops?

- Do you think this changed over time?
- If not, what do you think might have prevented people from engaging with the workshops?

1. Are you aware of the intervention toolkit?

- During the workshops, did you see the toolkit emerging?
- Did your understanding of the task at hand develop/become clearer as the workshops progressed?

**Follow Up**

1. Did you enjoy the co-design process?

- What did/didn’t you enjoy about it?
- Following the workshops, were there any additional discussions about the co-design process?

1. Would you get involved in a similar project again in the future?

- If so why/why not?

1. Do you feel you benefited from participating in the workshops? If so, how?
2. Do you feel that your participation in the process has impacted your team? If so, how?
3. What elements of the co-design process would you have discussed with your colleagues?
4. Do you think your participation has had an impact on your organisation? If so, how?
5. Following the workshops, has participating in the co-design process changed your day-to-day work in anyway?
6. Any additional thoughts on how the process could have been improved?
7. Is there anything else you would like to add about your experience that I haven’t asked about?

{Thank participant for their time; ask if they would like to see results when study is completed}
