# Supplementary material for: A qualitative evaluation of participants' experiences of using co‐design to develop a collective leadership educational intervention for health‐care teams
Source: Health Expect. 2020 Jan 30;23(2):358–67. doi: 10.1111/hex.13002 (PMC7104638; doi:10.1111/hex.13002)
Supplement: Supplementary file 3 [file HEX-23-358-s003.docx]

**Appendix C – Workshop feedback form**

1. Was it worth attending? Yes/No? Why?
2. Do you think we have made progress? Yes/No? Why?
3. Do you understand how the components of today’s workshop fit with the co-design process? *(Only workshops 2-6)*
4. Do you understand the aims of the Co-Lead programme and the purpose of the co-design phase? Yes/No? Why? *(Only workshop 1)*
5. Was there any aspect of today’s workshop that worked well/did not work well? *(Only workshops 2-6)*
6. What is your key take home message?
7. Do you have any additional comments or any comments on the venue, timing, catering, etc.?
